# Radon Solubility in Different Tissues after Short Term Exposure

**DOI:** 10.3390/ijerph20031773

**Published:** 2023-01-18

**Authors:** Annika Hinrichs, Michaela Schmitt, Franziska Papenfuß, Mirjam Roth, Claudia Fournier, Gerhard Kraft, Andreas Maier

**Affiliations:** 1 GSI Helmholtzzentrum für Schwerionenforschung GmbH, 64291 Darmstadt, Germany; 2 Physics Department, Goethe University Frankfurt am Main, 60438 Frankfurt, Germany; 3 Chemistry and Biotechnology Department, University of Applied Science, 64295 Darmstadt, Germany

**Keywords:** radon, solubility, tissue, distribution

## Abstract

Radon, a naturally occurring radioactive noble gas, contributes significantly to lung cancer when incorporated from our natural environment. However, despite having unknown underlying mechanisms, radon is also used for therapeutic purposes to treat inflammatory diseases such as rheumatoid arthritis. Data on the distribution and accumulation of radon in different tissues represent an important factor in dose determination for risk estimation, the explanation of potential therapeutic effects and the calculation of doses to different tissues using biokinetic dosimetry models. In this paper, radon’s solubility in bones, muscle tissue, adipose tissue, bone marrow, blood, a dissolved gelatin and oleic acid were determined. In analogy to current radon use in therapies, samples were exposed to radon gas for 1 h using two exposure protocols combined with established γ-spectroscopic measurements. Solubility data varied over two orders of magnitude, with the lowest values from the dissolved gelatin and muscle tissue; radon’s solubility in flat bones, blood and adipose tissue was one order of magnitude higher. The highest values for radon solubility were measured in bone marrow and oleic acid. The data for long bones as well as bone marrow varied significantly. The radon solubility in the blood suggested a radon distribution within the body that occurred via blood flow, reaching organs and tissues that were not in direct contact with radon gas during therapy. Tissues with similar compositions were expected to reveal similar radon solubilities; however, yellow bone marrow and adipose tissue showed differences in solubility even though their chemical composition is nearly the same—indicating that interactions on the microscopic scale between radon and the solvent might be important. We found high solubility in bone marrow—where sensitive hematopoietic cells are located—and in adipose tissue, where the biological impact needs to be further elucidated.

## 1. Introduction

The radioactive noble gas radon (^222^Rn) contributes up to half of radiation exposure from natural sources [1]. There is consent that environmental exposure to radon and its progeny is classified as carcinogenic for lung cancer [2]. It is responsible for around 2 % of all cancer deaths in Europe and increases the risk of lung cancer by 16 % per 100 Bq/m^3^ radon [3,4]. However, radon is also used in inflammatory disease medical therapies via bathing in radon-containing water or radon inhalation [5]. Radon is produced within the uranium decay series, starting with ^238^U. A section beginning with ^226^Ra and ending with ^210^Pb is shown below:

Ra226 →∝ Rn222 →α Po218 →α Pb214 →β−,γ Bi214 →β−,γ Po214 →α Pb210

This decay chain is governed by the relatively long half-life of Rn222, at 3.8 d—the longest half-life of all radon isotopes. The α-emitters of this decay chain contribute the most to the total dose, whereas β- and γ-decay are only responsible for less than 10% [6]. Considering the short half-lives of the solid radon progeny—below 30 min (^218^Po: 3.098 min, ^214^Pb: 27.06 min, ^214^Bi: 19.9 min, ^214^Po: 163.3 μs)—and their initial charge directly after decay, they are likely to bind at their origin of formation and decay before clearance can occur [7]. Therefore, mainly primary radon is distributed throughout the body, and radon solubility is the major parameter determining its local distribution within different tissues.

For applications such as the above-mentioned therapies, a reliable risk assessment is indispensable for a balanced risk-to-benefit evaluation. Calculations of radon dose are mostly based on epidemiological data or biokinetic dosimetry models [8]. Besides morphometric and physiological parameters, these models depend on existing data on the diffusion and solubility of radon in different tissues [9,10,11], which are derived from animal experiments in rats [12] and, more recently, with mice [13]. In the present study, we provide new data on radon solubility in different biologically relevant materials and tissues, which can serve as input parameters for biokinetic dosimetry models, leading to a more accurate risk assessment. For the solubility experiments, different samples were selected with respect to their impact on risk assessment and their potential importance in the biological mechanisms of radon therapies. In these samples, the solubility of radon after one hour of exposure was determined. These datasets are needed in order to calculate organ- and tissue-to-blood partition coefficients, defined as the ratio of the radon concentration in tissue to the radon concentration in blood [10].

## 2. Materials and Methods

Experiments were performed in a radon chamber under controlled conditions (radon activity concentration, temperature and relative humidity) [14]. Samples were exposed at room temperature (295.5 ± 0.5 K) and atmospheric pressure (1001 ± 7 mbar) for one hour. Unless stated elsewhere, values are given as averages with their respective standard deviation and a confidence interval of 1σ. During experiments, the radon activity concentration was kept constant. As solubility was expected to vary between samples, the radon activity concentration (c(Rn)) was chosen in order to be able to record gamma spectra with count rates significantly higher than the background level. Therefore, samples with an anticipated high solubility were exposed to lower levels of radon (193 ± 16 kBq/m^3^) and samples with low solubility to high levels (6280 ± 230 kBq/m^3^), respectively.

The exposure time was chosen in accordance with how radon therapies are usually applied, in daily fractions of one hour. In earlier studies, Nussbaum and Hursh determined the accumulation of radon in fatty acids [15]. We used oleic acid (C_18_H_34_O_2_), which is the most common fatty acid in the human body [16], as the simplest model for fat cells in order to set up and evaluate an improved measurement protocol. As the composition of most common fatty acids in human and porcine adipose tissue is comparable [17], porcine fat is an appropriate model for representing human adipose tissue—with not only fatty acids, but also complete fat cells. However, under ambient exposure conditions or during therapy, adipose tissue is not in direct contact with radon. A direct interaction between radon and tissues only takes place in the lungs and on the skin. In order to estimate how much radon reaches different tissues and organs via blood flow, solubility in porcine blood was determined, as red blood cells in pigs and humans in particular share similarities [18]. Reaching the tissue, the total amount of radon remaining in the body depends on its solubility and on the tissues’ mass. Consequently, porcine muscle tissue was used as another sample, because the skeletal muscles contribute the most to the total body mass, with a share of around 40% [19]. The amount of radon dissolving within different tissues is not only important for risk assessments but is also important for understanding the potential molecular mechanisms of radon therapy. It is known that in musculoskeletal diseases, bones and parts of the joints are affected by erosion and resorption. Accumulated radon and its progeny at these body sites might be able to modulate inflammation [7] and, consequently, their solubility in bone marrow, cartilage and bones is of special interest; bone marrow in particular may contribute significantly, due to its composition. In addition, bone marrow has a high proportion of adipose tissue that increases with age [20]—which is then referred to as yellow bone marrow—where radon could possibly accumulate. On the other hand, the so-called red bone marrow, which is hematopoietic-active and could influence inflammation, is of special interest in risk assessments. Therefore, cattle bone marrow was separated from long bones (femur) and visually divided into red and yellow bone marrow. Marrowbones, as a representative of long bones, and ribs as representative of flat bones, were additionally exposed to radon. In order to expose the bones, the remaining marrow and other tissues were removed and the bones crushed into smaller pieces. As a model representing cartilage, a dissolved gelatin (50 mass percent gelatin in demineralized water) with more collagen than physiological levels [21] was chosen, in order to identify any potential effects of collagen on radon solubility.

**Figure 1:**
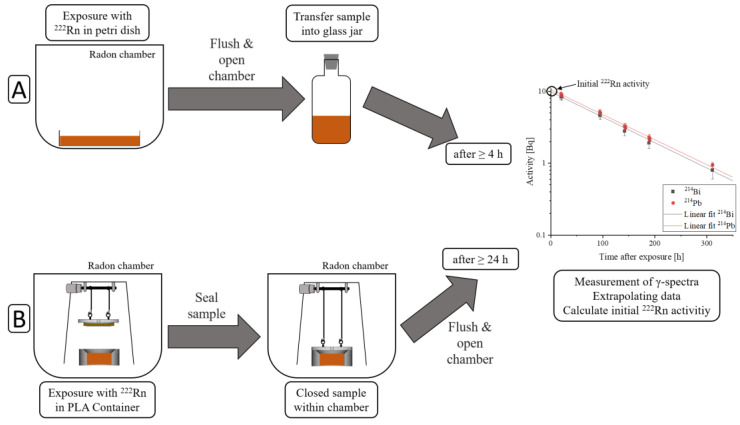
Comparison of the two exposure protocols. Part A describes the first, and part B the second protocol. In the first protocol, samples were exposed in the radon chamber in petri dishes to radon for 1 h at room temperature and atmospheric pressure. Afterwards, the chamber was flushed with clean air for five minutes. Then, the samples were transferred into a glass jar and sealed radon-tight. First measurements started no earlier than 4 h after sample transfer. In the second protocol, samples were exposed in a PLA-container to radon at room temperature and atmospheric pressure. After 1 h of exposure, samples were sealed within the chamber, followed by flushing the chamber. Then, 24 h later, the measurements were started, as surface contamination of the sample container had dissipated below detection limit. The measurements were carried out in the same way in both protocols; γ-spectra were recorded for a period of up to 14 d. By extrapolating the data, the initial radon activity inside the sample could be determined. Normalizing to the mass of the sample and the radon activity concentration during exposure enabled the calculation of radon solubility in the sample.

Samples were exposed to radon for one hour using two different protocols. The two different protocols used are schematically described in Figure 1, where exposure and sample sealing are different, but measuring the sample’s activity is the same in both protocols.

The first protocol proceeded as follows:

1. Exposure of samples to radon within a petri dish covered with a glass fiber filter to avoid contamination from radon progeny accumulating on the sample. They were positioned in order to prevent contact between the individual cuboid parts of the same sample. The surface-to-volume ratio in liquid samples was only determined by the sample’s height, whereas in solid samples, it was dependent on the sample’s geometry.
2. After one hour of exposure, the radon chamber was flushed with clean air for five minutes to remove the radon gas, for safety.
3. The radon chamber was opened, the samples were transferred into an uncontaminated glass jar and sealed radon-tight.
4. No earlier than four hours later, measurements of activity could be started. This waiting time was necessary to reach a radioactive equilibrium between radon and its γ-emitting progeny (^214^Pb and ^214^Bi), in order to be able to calculate the radon activity by measuring ^214^Pb and ^214^Bi activity.

The second protocol, using a new setup, was recently developed to enable the measurement of the amount of radon dissolved over time. This improvement was implemented by sealing the samples within the radon chamber in order to minimize radon diffusion out of the sample, while flushing the chamber with clean air. The sample containers were designed in a way that excluded air when closing them. The procedure was as follows:

1. Samples were exposed to radon within 3D-printed PLA containers (C_3_H_4_O_2_). These containers can be designed individually in order to fulfil particular requirements. By varying the sample’s containers height and, consequently, the sample’s height, it is easily possible to change the surface-to-volume ratio.
2. After one hour of exposure, the containers were automatically sealed within the radon chamber with the new setup.
3. The radon chamber was flushed with clean air for five minutes for safety.
4. The radon chamber was opened and the radon-tight sealing of the sample containers was checked.
5. At least 24 h after exposure, surface contamination of the sample container surface due to radon progeny had dissipated below the detection limit, and measurements of its activity could be started.

After exposure, radon activity within the samples was calculated from the γ-radiation of the radon progeny (^214^Pb and ^214^Bi). First measurements were started no earlier than four hours after exposure, when a radioactive equilibrium between radon and its progeny was achieved—resulting in identical activities of ^222^Rn, ^214^Pb and ^214^Bi. With a high purity germanium-detector (BE3825, Mirion Technologies GmbH, Rüsselsheim, Germany), γ-spectra were recorded for a period of up to 14 days. γ-spectra were analyzed using a commercially available software (Genie2000, Mirion Technologies GmbH, Rüsselsheim, Germany). As a first step, energy calibration was performed using the most abundant decay energies of ^214^Pb (242 keV, 295 keV, 352 keV) and ^214^Bi (609 keV). In the next step, an efficiency calibration was performed, considering the sample geometry, sample density and detector properties with ISOCS/LabSOCS calibration software (Mirion Technologies GmbH, Rüsselsheim, Germany). For this, the detector was characterized at the factory, and afterwards, the efficiency calibration was calculated with an internal model. Finally, the activity was calculated, considering the branching ratio, the measurement time and the radioactive decay during measurements. Various errors during these steps were processed within the software and included in the error of the calculated activity. A more detailed explanation can be found in [6].

By plotting the measured ^214^Pb and ^214^Bi activities over time after exposure and extrapolating the data, the intersection with the *y*-axis corresponds to the initial radon activity (A_Sample_). When the sample’s activity was still higher than background, a measurement period that covered more than three half-life periods was chosen in order to improve the extrapolated fit. A detailed description of the measurement protocol can be found in [22]. The ratio of the calculated initial radon activity in each sample with its mass (m_Sample_) yielded the solubility. This was normalized to the radon activity concentration during exposure (c(Rn)_Chamber_). The solubility (S) measured here refers to the amount of radon solving within one hour of exposure, which is equivalent to therapy conditions. Errors for solubility were calculated using Gaussian error propagation. The equation for the solubility calculation can be described as follows:

$$S=\frac{\frac{A_{\mathrm{Sample}}}{m_{\mathrm{Sample}}}}{c{\left(\mathrm{Rn}\right)}_{\mathrm{Chamber}}}$$

In order to determine solubility after 1 h of exposure, food-quality fresh bones, muscle tissue and adipose tissue were bought. These solid samples, as well as dissolved gelatin, were cut and bones were crushed into pieces with a maximum size of 1 × 2 × 3 cm and placed in petri dishes. These samples and half of the experiments with oleic acid were conducted using the first protocol. It is necessary to expose bone fragments following the first protocol, as placing these fragments into PLA containers and sealing them within the chamber would result in an inhomogeneous sample distribution with airspaces in-between. This would lead to an inclusion of air containing radon and the measured activity would be a sum of the radon in the bones and in the air, and therefore would result in higher measured activities.

The second protocol was used to measure radon solubility in blood and red and yellow bone marrow as well as half of the oleic acid samples. The exposure details of the different samples are shown in Table 1. In order to be able to compare the results obtained with the two different protocols, radon solubility in oleic acid was measured with both protocols and compared afterwards.

## 3. Results

**Figure 2:**
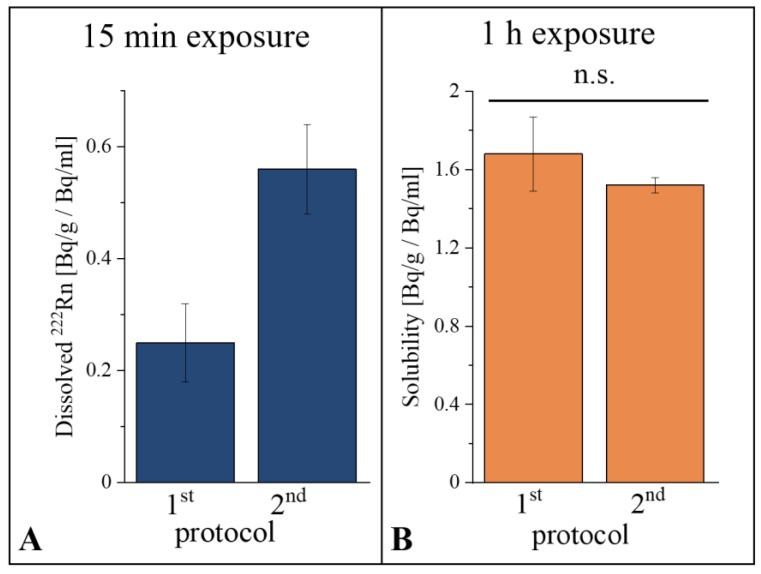
(**A**) Comparison of dissolved radon in oleic acid exposed to radon for 15 min with the first and second exposure protocols. The experiment was performed at a temperature of 295.7 ± 0.1 K and an air pressure of 1001 ± 3 mbar, with a radon activity concentration of 287 ± 22 kBq/m^3^. Values determined with the second setup showed about a factor of two-higher solubility. (**B**) Comparisons of the solubility data for oleic acid exposed to radon for 1 h obtained using the first and second exposure protocol. The solubility results using the two different protocols showed no significant difference. Experiments were performed at a temperature of 295.5 ± 0.4 K and at an air pressure of 1000 ± 9 mbar (with standard deviation). n.s.: not significant.

Measurements were first performed in order to validate the experimental improvements, using the second exposure protocol with the recently built setup. Moreover, it had to be tested whether the results obtained with the two different protocols were comparable. Therefore, oleic acid was exposed to radon for 15 min according to both protocols. Exposing samples following the second protocol increased the obtained amount of dissolved radon by around a factor of two (Figure 2A), demonstrating the improvement of the second setup combined with the second procedure, where radon loss due to diffusion is reduced. For short exposure times with an appropriately lower amount of dissolved radon compared to saturation, it is crucial to reduce the diffusion of radon out of the sample to a minimum while flushing the radon chamber with clean air. Therefore, the second measurement protocol guarantees a more accurate quantification of dissolved radon before saturation is reached. After demonstrating the improvement of the second setup, oleic acid was exposed to radon following both protocols for one hour in order to reach a saturation of radon in the sample, and the solubility data were compared (Figure 2B). These solubility values showed no significant differences, leading to the conclusion that the solubility results determined using the two different protocols after 1 h of exposure were comparable. As such, the majority of the radon had penetrated into the deeper layers and would not diffuse significantly during the short sample transfer period. Accordingly, it is not necessary to assign the results to a specific exposure protocol if samples are exposed for only one hour.

Subsequently, the radon solubility in the samples was determined. By normalizing the activity to the applied radon activity concentration, the measured solubility was independent of it. The radon solubility in oleic acid and in the dissolved gelatin when exposed to different activity concentrations is shown in Table 2.

**Figure 3:**
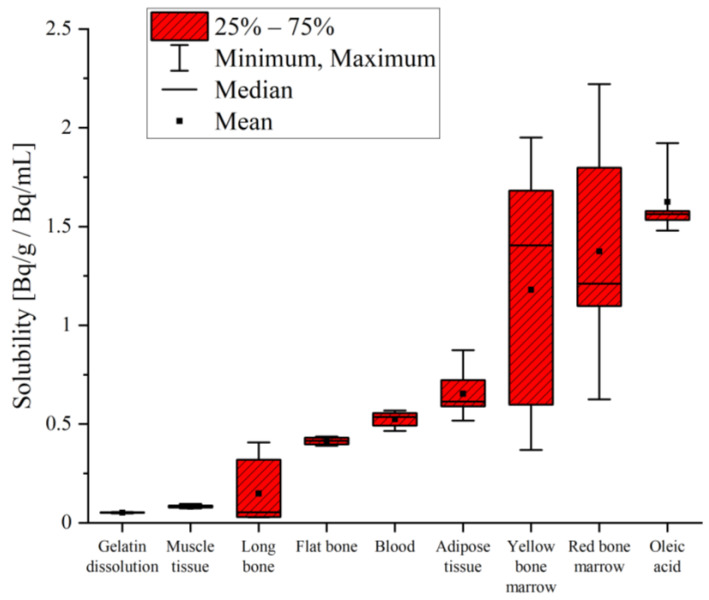
Radon solubility measured in different samples and at different radon activity concentration during the experiment. Experiments were performed at a temperature of 295.5 ± 0.5 K and at an air pressure of 1001 ± 7 mbar (with standard deviation). Bone, dissolved gelatin, muscle tissue and adipose tissue data were obtained using the first exposure protocol. Data for blood and bone marrow were determined with the second exposure protocol. Oleic acid was exposed and measured using both protocols.

The solubility varied over two orders of magnitude between the samples. Radon in the dissolved gelatin and muscle tissue from a pig showed the lowest solubility, with values below 0.1 Bq/g/Bq/mL. One order of magnitude-higher solubility was measured in the bones, blood and adipose tissue. The highest solubility values were measured in the yellow and red bone marrow, as well as in the oleic acid. The results for radon solubility are shown in Table 3 and a comparison is additionally presented in Figure 3.

## 4. Discussion

Solubility data were obtained using two different exposure protocols, including a specialized one for low concentrations and short exposure times that minimizes radon loss during transfer. Radon loss became especially important for short exposure times where only the proximal sample layers were radon-contaminated (Figure 2A). With long exposure times, radon penetrated into the deeper layers of the sample, and therefore loss from the inner layers was not very likely. The results for 1 h exposure were comparable for both protocols (Figure 2B).

Analogously to currently used therapy protocols, samples were exposed to radon for 1 h. Even though saturation of radon inside the samples was not yet reached, determining radon solubility at saturation would be of interest; however, tissue samples could not be exposed for much longer than one hour, as keeping them at room temperature for longer times would lead to further degradation at the cellular level. These microscopic changes would possibly influence the radon’s solubility, making longer exposure times pointless. Additionally, cooling the samples during exposure would increase the solubility [15,23]. Therefore, the determination of solubility at saturation in tissue samples is not possible with the present experimental setup. The presented results provide therapy-compatible information on the degree of radon accumulation in different organs and tissues. This is of great interest for the potential anti-inflammatory effects of radon as it used in therapy, but also for risk estimations for different exposure times [7].

Similar to our study, Nussbaum and Hursh [15] measured the radon solubility in oleic acid. Using a density of 0.895 g/mL for this specimen, they obtained a solubility of 9.05 Bq/g/Bq/mL, which is more than a factor of five-higher compared to the results presented here. However, in their study, the samples were exposed for 2 h and were rotated the whole time, resulting in a saturated sample.

Nussbaum/Hursh [12] and Ishimori et al. [13] experimentally determined the radon solubility in different samples in rats and mice, respectively, but only blood, muscle tissue and fatty tissue were also exposed in our study (Table 4). In all this literature, solubility values were gained after saturation was reached, which was not the case in most of our tissues.

**Figure 4:**
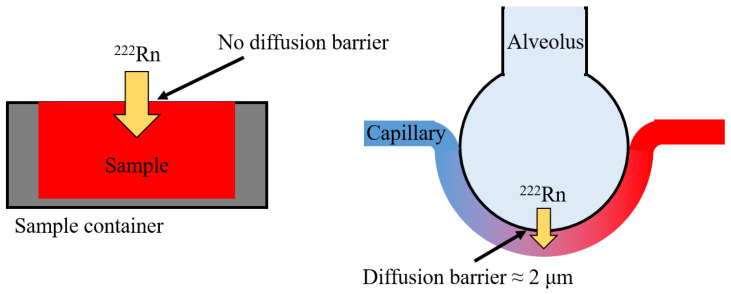
Schematic drawing of exposure scenarios. Left side shows in vitro sample exposure with no diffusion barrier, as performed in this study; right side shows in vivo diffusion within lungs with a diffusion barrier of around 2 μm, as seen in animal studies.

In Table 4, only the radon solubility in blood is the same order of magnitude, but is approximately 20% higher than reported in the literature—even though the exposure protocols differed significantly. In the in vitro experiments, blood was exposed in containers, while in the in vivo experiments, radon interacted with blood distributed in capillaries in the lung (Figure 4). With an air–blood barrier thickness of on average 2 μm in the human lung, the diffusion length is minimal, and saturation—depending on the partition coefficient between the air and blood—is supposed to be reached instantly [1]. During the blood sampling period in the animal studies, radon can diffuse out of the blood, explaining the higher values observed in our data.

In experiments performed by Nussbaum/Hursh and Ishimori et al., living animals were exposed to radon, resulting in a different mechanism of distribution. Within a living organism, radon dissolves in the blood, is actively distributed by the blood flow and diffuses out of the blood into different organs and tissues. In contrast, in our study, there was a direct contact surface between radon in the air and the tissues, and radon was transported into deeper layers by diffusion only. Active transport into the tissue combined with longer exposure times probably led to the saturation of radon within the different samples. This explains our lower solubility data compared to in vivo measurements (Table 4). Additionally, Sakoda et al. [24] showed that saturation in muscle tissue is reached faster than in adipose tissue. Therefore, we expect the observed solubility in muscle tissue to be closer to the literature data compared to that in the adipose tissue. While our data in muscle tissue was only about a factor of two smaller, in adipose tissue, the difference was by more than a factor of five.

**Figure 5:**
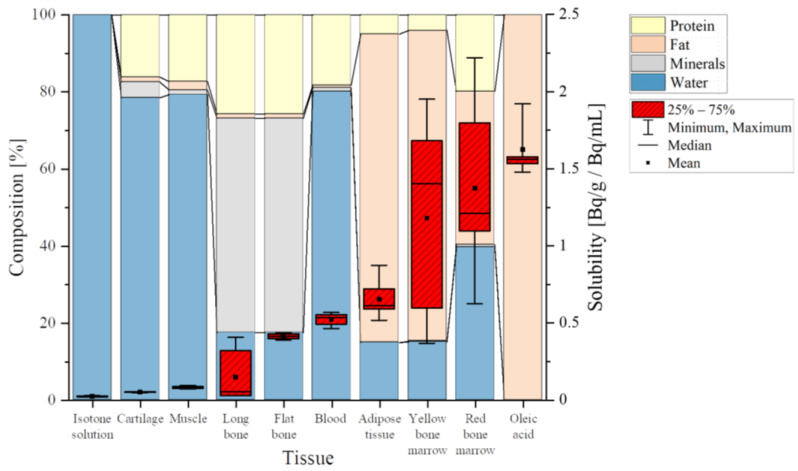
Solubility data (red boxes) and tissue composition divided into protein (yellow), fat (orange), minerals (grey) and water (blue) for different samples and tissues; data for tissue composition obtained from (14), same data for long and flat bones.

The main important topic of our study was the influence of the molecular composition of tissues. In Figure 5, solubility data (red boxes) are compared, with the chemical composition of different samples grouped into protein (yellow), fat (orange), minerals (grey) and water (blue)—obtained from [19]—where bone composition was not further subdivided, similar to ICRP 23.

These data were grouped according to increasing radon solubility and compared to their chemical composition, with the expectation that solubility would be additively determined by the various components; decreasing water content and increasing fatty components should increase the solubility of the samples. Our results matched this expectation in part. Cartilage, muscle tissue and blood are very similar in their composition, with a high proportion of water (around 80%) and only small amounts of fat (around 2%). Considering the minimal radon solubility in isotone solution (deionized water + 0.9% NaCl) [22], similar values were expected and were confirmed for cartilage and muscle tissue (Table 5). However, in blood, which consists of a similar amount of water, radon dissolved by about a factor of 10 more—indicating a more complex correlation between composition and radon solubility than plain additivity.

This complex behavior again became evident when comparing adipose tissue and yellow bone marrow—both having a high share of fat (80%) and similarities in their characteristics [25], suggesting comparable solubility. However, experimentally, the mean radon solubility in yellow marrow was nearly twice as high as in adipose tissue, but with a great variance in yellow bone marrow in the range of 0.37 to 1.95 Bq/g/Bq/mL (Table 3). Another argument against plain additivity based on composition was found in red bone marrow; although it is made up of 40 % of water, its mean solubility was 1.37 ± 0.48 Bq/g/Bq/mL—the second highest solubility—with variations between 0.62 and 2.22 Bq/g/Bq/mL. One reason for these discrepancies could be interindividual variations in bone marrow composition [26]. Additionally, the separation process of the bone marrow was based on optical appearance and needed to be optimized. In particular, the sample categorized as red bone marrow might contain yellow marrow mixed with blood, which then optically appears similar to red bone marrow.

Bones show a completely different composition compared to any other tissue or sample measured within this study. Bones are characterized by a low proportion of fat (1%), a small amount of water (17%) and a high share of minerals—at 54% of their total mass [19]. During bone preparation, complete tissue removal was not technically possible—especially in long bones, where remaining bone marrow might have changed the solubility values. Tissue residues in general cause a wider range of values measured in bones. The observed discrepancies between flat and long bones could be due to their differences in composition. In total, ICRP displays the chemical composition for bones and does not differentiate between different types, as shown in Figure 5. However, they present additional data comparing two single-skeleton analyses. These data not only show differences in the composition of tibia and ulna (long bones) bones compared to ribs (flat bones) but, additionally, a variability of the same bone between different individuals of the same species becomes obvious [19].

## 5. Conclusions

In summary, radon solubility was measured in a large variety of tissue and organic samples. In these measurements, it was expected that decreasing water content and increasing fat content would increase the radon solubility in the samples. The results for the dissolved gelatin, muscle and adipose tissue as well as the yellow bone marrow supported this assumption. However, the solubility obtained in the bones, blood and red bone marrow contradicted this hypothesis—indicating that the solubility of radon, which has no chemical interactions, does not show additive effects depending on the chemical composition of the tissue, but is affected by their microscopic structure. This finding has to be studied in greater detail in order to provide solid data for radiation protection and therapies. For example, Khursheed [10] calculated solubility in red bone marrow and in breast on the basis of the solubility obtained in adipose and soft tissue, weighted by their contribution to the tissue. This weighted average was mainly based on data obtained by Nussbaum and Hursh [12] in rats. However, it was then implemented in model calculations to determine doses in different tissues, which might be further elucidated in the future.

## Figures and Tables

**Table 1:** Exposure details of different samples including the used exposure protocol, the range of the sample’s mass, its geometry and the number of samples. The cubic geometry is characterized by the height, width and length, and the cylinders by their maximum radius (r_max_) and maximum height (h_max_).

Sample	Exposure Protocol	Mass [g]	Geometry, Maximum Size [cm]	Number of Samples
Long bones	1st	46.1–89.0	Cuboid, 1 × 2 × 3	3
Flat bones	1st	20.7–25.3	Cuboid, 1 × 2 × 3	2
Muscle tissue	1st	18.5–67.4	Cuboid, 1 × 2 × 3	4
Adipose tissue	1st	16.7–26.5	Cuboid, 1 × 2 × 3	5
Dissolved gelatin	1st	45.6–81.3	Cuboid, 1 × 2 × 3	4
Oleic acid	1st and 2nd	12.0–28.8	Cylinder, r_max_ = 3.1, h_max_ = 1	6
Blood	2nd	30.0	Cylinder, r = 3.1, h = 1	3
Red bone marrow	2nd	2.5–2.9	Cylinder, r = 1.5, h = 0.5	8
Yellow bone marrow	2nd	2.6–9.4	Cylinder, r_max_ = 2.5, h = 0.5	19

**Table 2:** Comparison of solubility data obtained by the exposure of samples to different radon activity concentrations (c(Rn)) and subsequent normalization to the according activity concentrations.

Oleic Acid		Dissolved Gelatin	
c(Rn) [kBq/m^3^]	Solubility [Bq/g/Bq/mL]	c(Rn) [kBq/m^3^]	Solubility [Bq/g/Bq/mL]
283 ± 6	1.53 ± 0.13	2840 ± 100	0.054 ± 0.006
3760 ± 100	1.58 ± 0.05	6280 ± 230	0.052 ± 0.003

**Table 3:** Radon solubility in different samples.

Sample	Radon Solubility [Bq/g/Bq/mL]
Dissolved gelatin (50 mass percent)	0.051 ± 0.003
Muscle tissue	0.081 ± 0.008
Long bones	0.15 ± 0.17
Flat bones	0.42 ± 0.02
Blood	0.53 ± 0.04
Adipose tissue	0.65 ± 0.12
Yellow bone marrow	1.18 ± 0.55
Red bone marrow	1.37 ± 0.48
Oleic acid	1.62 ± 0.17

**Table 4:** Radon solubility data obtained in this study compared to literature data from Nussbaum/Hursh [12] and Ishimori et al. [13].

Tissue	Radon Solubility [Bq/g/Bq/mL]		
	This Study	Nussbaum/Hursh	Ishimori et al.
Blood	0.53 ± 0.04	0.405 ± 0.016	0.410 ± 0.016
Muscle tissue	0.081 ± 0.008	0.154 ± 0.005	0.188 ± 0.052
Adipose tissue	0.65 ± 0.12	4.83 ± 0.07	3.730 ± 0.142

**Table 5:** Radon solubility in samples with a high proportion of water (>75%).

Sample	Radon Solubility [Bq/g/Bq/mL]
Isotone solution [22]	0.025 ± 0.003
Dissolved gelatin (50 mass percent)	0.051 ± 0.003
Muscle tissue	0.081 ± 0.008
Blood	0.53 ± 0.04

## Data Availability

The data presented in this study are available on request from the corresponding author.
